# Transforming Growth Factor-Beta1 in Diabetic Kidney Disease

**DOI:** 10.3389/fcell.2020.00187

**Published:** 2020-03-24

**Authors:** Lijun Zhao, Yutong Zou, Fang Liu

**Affiliations:** Division of Nephrology, West China Hospital, Sichuan University, Chengdu, China

**Keywords:** diabetic kidney disease, transforming growth factor-β1, fibrosis, inflammation, Smad signaling

## Abstract

Diabetic kidney disease (DKD) is the leading cause of end-stage renal disease (ESRD) worldwide. Renin-angiotensin-aldosterone system (RAAS) inhibitors and sodium-glucose co-transporter 2 (SGLT2) inhibitors have shown efficacy in reducing the risk of ESRD. However, patients vary in their response to RAAS blockades, and the pharmacodynamic responses to SGLT2 inhibitors decline with increasing severity of renal impairment. Thus, effective therapy for DKD is yet unmet. Transforming growth factor-β1 (TGF-β1), expressed by nearly all kidney cell types and infiltrating leukocytes and macrophages, is a pleiotropic cytokine involved in angiogenesis, immunomodulation, and extracellular matrix (ECM) formation. An overactive TGF-β1 signaling pathway has been implicated as a critical profibrotic factor in the progression of chronic kidney disease in human DKD. In animal studies, TGF-β1 neutralizing antibodies and TGF-β1 signaling inhibitors were effective in ameliorating renal fibrosis in DKD. Conversely, a clinical study of TGF-β1 neutralizing antibodies failed to demonstrate renal efficacy in DKD. However, overexpression of latent TGF-β1 led to anti-inflammatory and anti-fibrosis effects in non-DKD. This evidence implied that complete blocking of TGF-β1 signaling abolished its multiple physiological functions, which are highly associated with undesirable adverse events. Ideal strategies for DKD therapy would be either specific and selective inhibition of the profibrotic-related TGF-β1 pathway or blocking conversion of latent TGF-β1 to active TGF-β1.

## Introduction

Diabetic kidney disease (DKD), the most common cause of end-stage renal disease (ESRD) worldwide, accounts for about 40% of new cases of ESRD each year in the United States and China (Zhang et al., 2016; Alicic et al., 2017). With the increasing incidence of diabetes, there is a heightened need for therapy to delay progression of DKD. Existing therapies have had limited success. Renin-angiotensin-aldosterone system (RAAS) inhibitors, such as losartan and irbesartan, have been effective in reducing the risk of ESRD for patients with DKD (Brenner et al., 2001; Lewis et al., 2001; Parving et al., 2001). However, patients exhibited great variation in their responses to RAAS blockades. In the past two decades, there has been a decline in the rate of acute myocardial infarction and death from hyperglycemic crisis, but no change has occurred in the rate of ESRD (Gregg et al., 2014). Although sodium-glucose co-transporter 2 (SGLT2) inhibitors have conferred cardiovascular and renal protection (Perkovic et al., 2019), effective therapy for DKD is still unavailable. An epidemiological study revealed that the 5-year mortality rate of DKD was approximately 40%, as high as many cancers (Abdel Aziz et al., 2017). Transforming growth factor-β1 (TGF-β1) signaling contributes to DKD progression, and inhibiting TGF-β1 signaling has shown potential renoprotective properties in animal and human studies. In this mini-review, we discuss the pleiotropic and the potential therapeutic effects of TGF-β1 in DKD.

## Tgf-β1 and Tgf-β1 Signaling Pathway

TGF-βs exist as five isoforms, but only TGF-β1, TGF-β2, and TGF-β3 are present in mammals; the three isoforms elicit similar responses *in vitro*. TGF-β1, the most abundant isoform, is synthesized by all types of resident renal cells and infiltrating inflammatory cells (Aihara et al., 2010). TGF-β1 is secreted into the extracellular matrix (ECM) in an inactive complex (latent TGF-β1) with TGF-β-latency-associated peptide (LAP) and latent TGF-β binding proteins (LTBP) (Munger et al., 1997). The activation of latent TGF-β1 is mediated by proteolytic cleavage in the presence of the serine protease plasmin, reactive oxygen species (ROS), thrombospondin-1 (TSP-1), or integrins (Khalil, 1999; Kim et al., 2018). Integrins bind to the arginine-glycine-aspartic acid sequence in LAP. This binding appears to change the conformation of the latent TGF-β1 complex by tractional force (Munger et al., 1999). This conformational change presents the latent TGF-β1 complex to transmembrane metalloproteinases, such as membrane-type-1-matrix metalloproteinase (MT-1-MMP), which cleave the latent TGF-β1 complex and release active TGF-β1 (Mu et al., 2002; Sheppard, 2004; Araya et al., 2006; Wipff and Hinz, 2008). Active TGF-β1 interacts with its receptors to activate Smad-dependent and Smad-independent downstream signaling (Lan, 2011; Sutariya et al., 2016).

### Smad-Dependent Signaling Pathway

Active TGF-β1 binds to a Type II membrane receptor, TGF-β Type II receptor (TβRII). This binding results in the phosphorylation and recruitment of the TGF-β Type I receptor (TβRI). The activated complex of TGF-β1-TβRI-TβRII phosphorylates Smad2 and -3. Then, the phosphorylated Smad2 and -3 bind to Smad4 to form the Smad complex (Lan, 2011). This Smad complex translocates into the nucleus and binds to Smad-binding elements (SBEs) or Smads-containing complexes (Nakao et al., 1997b; Meng et al., 2013), in turn, regulating transcription of genes encoding, e.g., collagen, fibronectin, α-smooth muscle actin (Chakravarthy et al., 2018), and Smad7 (Yan et al., 2009).

Smad proteins are classified into three subgroups. Smad2 and -3 comprise the receptor-regulated Smads (R-Smads) for TGF-β1 signaling, and Smad1, -5, and -8 for bone morphogenetic protein (BMP) signaling. Smad2 and -3 are key downstream mediators of TGF-β1, and they are highly activated in animal renal tissues in DKD (Isono et al., 2002; Høj Thomsen et al., 2017). Smad2 and -3 may have distinct functions in renal fibrosis. Either a Smad3 knockout or a Smad3-specific inhibitor delayed de-differentiation of proximal tubular cells and alleviated renal fibrosis in a streptozotocin-induced model of diabetes (Fujimoto et al., 2003; Li et al., 2010). These findings suggested that TGF-β1/Smad3 signaling has critical activities in renal fibrosis. Conversely, unlike Smad3, the function of Smad2 in DKD is unclear. Overexpression of Smad2 attenuated TGF-β1-induced phosphorylated Smad3 and collagen expression, whereas deletion of Smad2 promoted renal fibrosis via substantially enhanced Smad3 signaling (Meng et al., 2010; Loeffler et al., 2018). Although Smad2 interacts with Smad3 physically, Smad2 and -3 may compete for phosphorylation in response to TGF-β1 stimulation. Thus, Smad2 may competitively inhibit phosphorylation of Smad3 in response to TGF-β1 (Meng et al., 2010). Besides TGF-β1 signaling, Smad2 nuclear translocation and phosphorylation can also be mediated by advanced glycation end-products in DKD (Li et al., 2004). Thus, the activity of Smad2 is complicated in DKD.

The second Smad subgroup is the common-partner Smad (co-Smad), Smad4, which forms a heterotrimeric complex with phosphorylated R-Smads. The Smad4-containing complex translocates into the nucleus and regulates expression of the genes indicated earlier. Furthermore, Smad4 is implicated in suppressing nuclear factor-κB (NF-κB)-driven inflammation by inducing Smad7 expression (Ka et al., 2012).

The third Smad subgroup is the inhibitory Smads (I-Smads). Members of this Smad family have a conserved carboxy-terminal MH2 domain. I-Smads inhibit TGF-β1 family signaling via interaction with type I receptors, and I-Smads compete with R-Smads for receptor activation (Miyazawa and Miyazono, 2017). Smad7, one of the most investigated I-Smad in DKD, can cause degradation of TβRI and Smads activity in a negative feedback process. Smad7 inhibits Smad2/3 during renal fibrosis. In chronic kidney disease, TGF-β1 signaling upregulated the Smurfs and caused ubiquitin-dependent degradation of Smad7, which led to a decrease in Smad7 protein level (Kavsak et al., 2000; Ebisawa et al., 2001; Fukasawa et al., 2004; Liu et al., 2008). Smad7 knockout mice progressed to more severe interstitial fibrosis and enhanced inflammation (Cheng et al., 2013; Chung et al., 2013), and overexpression of Smad7 in kidney was effective in reducing collagen matrix expression and in alleviating inflammatory infiltration in DKD (Ka et al., 2012). These findings revealed anti-fibrotic and anti-inflammatory functions of Smad7 in DKD.

### Smad-Independent Signaling Pathway

In addition to Smad-mediated transcription, TGF-β1 directly activates other signal transduction pathways in the pathophysiology of kidney disease. These other pathways include the mitogen-activated protein kinases (MAPK) pathway (Meng, 2019), growth and survival kinases phosphatidylinositol-3-kinase (PI3K)/Akt (Lu et al., 2019), small GTP-binding proteins such as Ras, RhoA, Rac1, and Cdc42, the Notch signaling pathway (Atfi et al., 1997; Sweetwyne et al., 2015), Integrin-linked kinase (ILK), and the Wnt/β-catenin pathway (Xu et al., 2017; Zhang and Huang, 2018). These non-canonical, non-Smad pathways can indirectly participate in de-differentiation of proximal tubular cells (Lu et al., 2019), apoptosis (Matoba et al., 2017), and matrix formation (Meng, 2019), thereby mediating signaling responses either as stand-alone pathways or as pathways that converge onto Smads to control Smad activities.

## Tgf-β1 Promotes Renal Fibrosis in DKD

Diabetic kidney disease pathology is characterized by thickening of the glomerular basement membrane, mesangial expansion, segmental glomerulosclerosis or global glomerulosclerosis, tubulointerstitial fibrosis, and afferent and efferent arteriole hyalinosis (Najafian et al., 2015). The TGF-β1 signaling pathway is activated in DKD, and the inhibition of TGF-β1 attenuates fibrosis in animal models of diabetes (Meng, 2019). Pathogenic stimuli in DKD activate TGF-β1 signaling. Angiotensin-II, which was elevated in mesangial cells and glomerular endothelial cells, has been implicated in activating TGF-β1 by generation of ROS from nicotinamide adenine dinucleotide phosphate oxidases (Lee, 2011; Morales et al., 2012) or by activating protein kinase C- and p38 MAPK-dependent pathways (Weigert et al., 2002). Hyperglycemia, mechanical stretch, and advanced glycation end products were found to upregulate TGF-β1 in DKD (Gruden et al., 2000; Chuang et al., 2015). TSP-1, a prototypic matricellular ECM protein, was heavily deposited in glomeruli of patients with DKD (Hohenstein et al., 2008). TSP-1 binds to the latent TGF-β1 complex, and, by a non-proteolytic mechanism, converts latent TGF-β1 to the active form, which leads to upregulation of TGF-β1 signaling (Murphy-Ullrich and Suto, 2018). Direct evidence for the importance of TSP-1 in regulating TGF-β signaling in DKD comes from two different models of type 1 diabetes. Streptozotocin-treated TSP-1 knockout mice showed decreased glomerular TGF-β signaling as measured by phosphorylated Smad2, and attenuated glomerulosclerosis (Daniel et al., 2007). In another type 1 diabetic animal model, uninephrectomized Akita mice treated with TSP-1 blocking peptide LSKL were protected from tubulointerstitial fibrosis and had reduced phosphorylation of Smad2 and -3 (Lu et al., 2011).

Mechanisms of TGF-β1 regulated fibrosis in DKD are multifactorial and involve (1) overexpression of ECM, (2) decreased degradation of ECM, (3) enhanced cross-linking between collagen and elastin fibers, and (4) overactivation of proximal tubular and endothelial cell de-differentiation. Both canonical TGF-β1/Smads-dependent signaling pathways and alternative signaling by TGF-β1 are involved in stimulating collagen expression and accumulation. Neutralizing all three mammalian TGF-β isoforms (-β1, -β2, and -β3) with antibodies reduced ECM gene (fibronectin and type IV collagen) expression and attenuated renal fibrosis in mice with type 1 or type 2 diabetes (Sharma et al., 1996; Ziyadeh et al., 2000). Thus, TGF-β1 has a critical signaling function in ECM accumulation in DKD.

TGF-β1 expression greatly inhibited ECM degradation by promoting the synthesis of plasminogen activator inhibitor-1 (PAI-1) which resulted in renal fibrosis (Shihab et al., 1997). The abundance of matrix metalloproteinase-9 (MMP-9), an ECM-degradation MMP, was decreased in transgenic mice that overexpressed TGF-β1 (Zechel et al., 2002; Ueberham et al., 2003). In addition, TGF-β1 augmented the expression of tissue inhibitor of metalloproteinases-1 (Ueberham et al., 2003; Abdel Aziz et al., 2017), which inhibited the ECM-degrading MMPs.

TGF-β1 promotes formation of the cross-linking between collagen and elastin fibers by upregulating lysyl oxidase (Boak et al., 1994; Di Donato et al., 1997). *In vitro*, TGF-β1 significantly increased (∼5 times) lysyl oxidase expression in tubular epithelial cells (Di Donato et al., 1997). In addition, TGF-β1 stimulated expression of procollagen lysyl hydroxylase 2, an enzyme that hydroxylates lysyl residues of collagen telopeptides and stabilizes collagen cross-linking (Gjaltema et al., 2015). Crosslinking increases ECM resistance to degradation by MMPs (El Hajj et al., 2018).

De-differentiation of the proximal tubular cells and endothelial cells contributes to renal fibrosis in diabetic mice. Extensive studies confirmed that TGF-β1 contributes to renal fibrosis by stimulating proximal tubular de-differentiation (Zeisberg et al., 2003) and endothelial de-differentiation (Li et al., 2009; Pardali et al., 2017). Hypoxia-inducible factor 1α (HIF-1α) accumulated in DKD and HIF-1α enhanced de-differentiation of murine proximal tubular epithelial cells *in vitro* (Higgins et al., 2007). Conditional HIF-1α ablation decreased interstitial collagen deposition and inhibited the development of tubulointerstitial fibrosis (Higgins et al., 2007). Although TGF-β1 stimulation increased HIF-1α expression, blocking TGF-β1 signaling inhibited HIF-1α activity, and, conversely, blocking HIF-1α activity decreased TGF-β1 signaling (Basu et al., 2011). These studies suggested cross-talk between TGF-β1 and HIF-1α signaling in regulating proximal tubular de-differentiation (Basu et al., 2011). As to endothelial de-differentiation, in animal models of folic acid nephropathy or unilateral ureteral obstruction, curtailed TGF-β signaling in the endothelium by endothelium-specific heterozygous TβRII knockout reduced endothelial de-differentiation and led to less tubulointerstitial fibrosis (Xavier et al., 2015). The mechanism by which TGF-β1 regulates endothelial de-differentiation is unknown. TGF-β1 stimulated endothelial de-differentiation in mouse endothelial cells by activating Snail expression (Kokudo et al., 2008).

**FIGURE 1:**
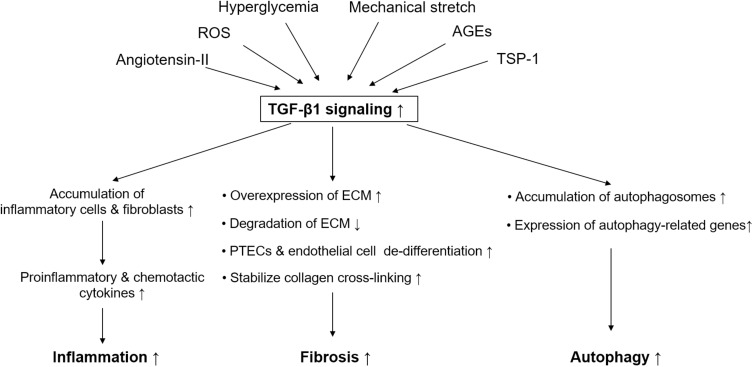
Simplified schematic diagram of pathological role of TGF-β1 signaling in diabetic kidney disease. Pathogenic stimuli in diabetic kidney disease like hyperglycemia, angiotensin-II, reactive oxygen species, mechanical stretch, advanced glycation end products, and thrombospondin-1 are able to active TGF-β1 signaling. TGF-β1 signaling plays an important role in mediating renal fibrosis, inflammation, and autophagy in proximal tubular epithelial cells in diabetic kidney disease. TGF-β, transforming growth factor-beta; ROS, reactive oxygen species; PTECs, proximal tubular epithelial cells; AGE, advanced glycation end products; TSP-1, thrombospondin-1; ECM, extracellular matrix.

In summary, the active TGF-β1 system promotes renal fibrosis, and it is involved in elevating collagen synthesis, suppressing ECM degradation, promoting collagen cross-linking, and fostering proximal tubular or endothelial cell de-differentiation (Figure 1).

## Diverse Inflammatory Functions of Tgf-β1 in DKD

TGF-β1 is a critical factor in the pathophysiological progression of DKD, having both pro- and anti-inflammatory properties (Sureshbabu et al., 2016).

TGF-β1 control of innate immune cells can have severe pathological consequences. Leukocytes and fibroblasts are recruited by the activation of resident kidney immune cells in DKD. This recruitment stimulates the expression of pro-inflammatory and chemotactic cytokines, which further drives the infiltration of monocytes and macrophages (Lv et al., 2018). TGF-β1 recruited macrophages and dendritic cells by stimulating the production of chemokines, including tumor necrosis factor-alpha (TNF-α), monocyte chemoattractant protein-1 (MCP-1), and inducible nitric oxide synthase. Furthermore, the secreted chemokines induced TGF-β1 expression in a positive feedback loop (Cheng et al., 2005), which sustained the high levels of TGF-β1 in the microenvironment. TGF-β1 induced the expression and release of other proinflammatory cytokines such as interleukin-8 (IL-8) and MCP-1 (Qi et al., 2006) in proximal tubular cells. In addition, TGF-β1 drove the differentiation of T helper 17 cells, which were activated in various proinflammatory conditions. In the presence of IL-6, TGF-β1 promoted the differentiation of naive T lymphocytes into proinflammatory T helper cells that produced IL-17 and augmented autoimmune conditions, which were enhanced by IL-1β and TNF-α (Korn et al., 2009; Sanjabi et al., 2009). In this way, TGF-β1 propagates and amplifies the proinflammatory and profibrotic processes that contribute to renal insufficiency in DKD (Figure 1).

Nevertheless, TGF-β1 also possesses anti-inflammatory properties, which was suggested by the findings that targeted deletion of the TGF-β1 gene resulted in profound multifocal inflammatory disease in mice (Shull et al., 1992). Additionally, TGF-β1 knockout mice developed severe inflammatory responses that were evidenced by massive lymphocytes, macrophages, immunoblasts, and plasma cell infiltration in many organs (Kulkarni et al., 1993). Tubular epithelial cell-specific TβRII knockout mice showed massive leukocytes or macrophages infiltration, increased proinflammatory cytokine release, and enhanced renal inflammation (Meng et al., 2012). Direct evidence for the importance of TGF-β1 in anti-inflammation comes from two studies. First, Ma et al. (2004) used animal studies to investigate the effect of different doses of TGF-β antibodies on glomerulosclerosis. Only low dose TGF-β antibody decreased macrophage infiltration, and reduced sclerosis, indicating that the amount of TGF-β may influence the inflammatory process. Second, regulatory T cells appeared to ameliorate DKD, and nude mice, which lacked all T-cell subtypes, had more severe DKD (Lim et al., 2010; Eller et al., 2011). In the presence of IL-2, TGF-β1 converted naive T cells into Foxp3 + regulatory T cells and inhibited the progression of DKD (Davidson et al., 2007; Kanamori et al., 2016).

Thus, the effects of TGF-β1 activation in renal inflammation may be protective or harmful depending on concentration or the presence of IL-6. However, the underlying mechanism by which TGF-β1 exerts its anti-inflammatory properties in DKD requires further investigation.

## Other Activities of Tgf-β1 in DKD

Recent studies illustrated that TGF-β1 promoted autophagy (Ding et al., 2010; Koesters et al., 2010). Autophagy, a system for removing protein aggregates and damaged organelles to maintain cellular homeostasis, is impaired in glomeruli and tubules in DKD (Yang et al., 2018). However, persistent activation of autophagy in kidney tubular epithelial cells induced tubular degeneration and promoted renal fibrosis (Livingston et al., 2016). Overexpression of TGF-β1 in renal tubules induced the accumulation of autophagosomes and stimulated expression of autophagy-related genes (Koesters et al., 2010; Xu et al., 2012). In proximal tubular cells, TGF-β1 promoted autophagy by generation of ROS, which contributed to the proapoptotic effect of TGF-β1 (Xu et al., 2012). Koesters et al. (2010) proposed TGF-β1-driven autophagy as a novel mechanism of tubular degeneration that led to renal interstitial fibrosis. On the contrary, TGF-β1 induced autophagy had positive effects. In a study by Ding et al. (2010), TGF-β1 induced autophagy in mesangial cells, and autophagy enhanced cell survival by preventing mesangial cells from undergoing apoptosis. Whether TGF-β1 driven autophagy has protective or deleterious effects on kidney depending upon the amount. In the study by Koesters et al., TGF-β1 level was higher than its level in pathological disease states, which triggered violent autophagy and promoted kidney injury. Thus, we need further clarification of the functions of TGF-β1 signaling-induced autophagy in the pathogenesis of DKD.

TGF-β1 also suppresses reabsorption of glucose by proximal epithelial cells. A dose-dependent increase in TGF-β1 expression by genetic manipulation increased urinary output of glucose in Akita mice, whereas genetic insufficiency of TGF-β1 decreased glucose output (Hathaway et al., 2015). Moreover, SGLT2 was directly regulated by TGF-β1 via Smad3 (Panchapakesan et al., 2013) and TGF-β1 showed decreased expression of SGLT1 and SGLT2 (Lee and Han, 2010). Thus, these results support the notion that TGF-β1 suppresses urinary glucose reabsorption in proximal tubular epithelial cells (Figure 1).

## Tgf-β1 Signaling as a Therapeutic Strategy for DKD

Blockade of TGF-β1 signaling as a therapeutic strategy has been achieved by gene technology and pharmacological drugs (Table 1). Inhibition of TGF-β with a pan-neutralizing monoclonal antibody (1D11) against all three isoforms ameliorated renal fibrosis and alleviated kidney structural changes in the rodent models of type 1 and type 2 diabetes mellitus (Sharma et al., 1996; Ziyadeh et al., 2000; Chen et al., 2003; Benigni et al., 2006). Pirfenidone is a low molecular weight synthetic molecule that has antifibrotic properties in animal models; it suppresses production of ROS and downregulates genes encoding profibrotic cytokines, such as α-SMA, collagen I, and collagen IV. Pirfenidone upregulates regulator of G-protein signaling 2 (Xie et al., 2016; Li et al., 2018; Pourgholamhossein et al., 2018). Moreover, RamachandraRao et al. (2009) found that pirfenidone decreased TGF-β promoter activity, blocked TGF-β1 production, and was effective in reducing mesangial matrix expansion and fibrosis in DKD. Switching TGF-β1 expression from low to high by genetic manipulation exacerbated renal injury in Akita mice, a result that further supported the idea that blockade of TGF-β1 was renoprotective for DKD (Hathaway et al., 2015).

**TABLE 1:** Pre-clinical and clinical studies aimed to TGF-β signaling in diabetic kidney disease.

Authors	Target	Method	Subject	Major findings
**Preclinical studies**				
Sharma et al., 1996	TGF-β1, TGF-β2, TGF-β3	Neutralizing monoclonal antibody	Streptozotocin-induced diabetic mice	Attenuated renal fibrosis
Ziyadeh et al., 2000	TGF-β1, TGF-β2, TGF-β3	Neutralizing monoclonal antibody	*db/db* mice	Decreased glomerular mesangial matrix expansion and attenuated renal fibrosis
Chen et al., 2003	TGF-β1, TGF-β2, TGF-β3	Neutralizing monoclonal antibody	*db/db* mice	Reversed the glomerular basement membrane thickening and mesangial matrix expansion, attenuated renal fibrosis
Benigni et al., 2006	TGF-β1, TGF-β2, TGF-β3	Neutralizing monoclonal antibody	Streptozotocin-induced diabetic mice	Alleviated sclerotic glomerulosclerosis and attenuated renal fibrosis
Petersen et al., 2008	TGF-β type I and type II receptor kinase activity	GW788388, pharmacological inhibitor	*db/db* mice	Decreased epithelial-mesenchymal transitions and attenuated renal fibrosis
RamachandraRao et al., 2009	TGF-β1 promoter activity; other pathways besides TGF-β (suppressing production of reactive oxygen species and downregulating profibrotic cytokine genes)	Pirfenidone, a pharmacological inhibitor	*db/db* mice	Ameliorated mesangial matrix expansion and attenuated renal fibrosis
Hathaway et al., 2015	TGF-β1	Genetic overexpression	Akita mice	Progressively exacerbated thicker glomerular basement membranes and severe podocyte effacement is dose-dependent
Fujimoto et al., 2003	Smad3	Genetic knockout	Streptozotocin-induced diabetic mice	Alleviated glomerular basement membrane thickness and attenuated renal fibrosis
Li et al., 2010	Smad3	SIS3, pharmacological inhibitor	Streptozotocin-induced diabetic mice	Attenuated renal fibrosis
Ka et al., 2012	Smad7	Ultrasound-mediated gene transfer of inducible Smad7 overexpression plasmids	*db/db* mice	Inhibited diabetic kidney injury including fibrosis and inflammation
Loeffler et al., 2018	Smad2	Renal tubular, endothelial, and interstitial cells-specific knockout	Streptozotocin-induced diabetic mice	Reduced epithelial-to-mesenchymal transition and attenuated renal fibrosis
**Clinical studies**				
Sharma et al., 2011	TGF-β1 promoter activity; other pathways besides TGF-β (suppressing production of reactive oxygen species and downregulating profibrotic cytokine genes)	Pirfenidone, a pharmacological inhibitor	Type 1 and type 2 diabetic patients	Increased estimated glomerular filtration rate level
Voelker et al., 2017	TGF-β1	Neutralizing monoclonal antibody added to renin-angiotensin-aldosterone system inhibitor	Type 1 and type 2 diabetic patients	Failed to slow the progression of diabetic kidney disease

*TGF-β1, transforming growth factor-β1.*

The success of TGF-β1 signaling inhibition in animal studies has promoted the strategy in clinical investigations with DKD (Sharma et al., 2011; Voelker et al., 2017). Pirfenidone significantly increased estimated glomerular filtration rates (eGFR) in a cohort of 77 diabetic patients with baseline eGFR of 20–75 ml/min/1.73 m^2^ (Sharma et al., 2011). However, a placebo-controlled, phase II study that used a humanized TGF-β1-specific neutralizing monoclonal antibody plus renin-angiotensin system blockades failed to slow the progression of DKD in diabetic patients who had eGFR of 20–60 ml/min/1.73 m^2^ (Voelker et al., 2017). Lack of improvement in clinical trials may be explained by the fact that rodent models of diabetes do not recapitulate tubulointerstitial fibrosis to the same degree observed in human disease. Also, inhibiting TGF-β1 fully and indiscriminately may not be wise because of its multiple physiological functions.

Nevertheless, targeting the conversion of latent to active TGF-β1 holds promise as a DKD therapeutic intervention. Animal studies revealed that overexpression of an active form of TGF-β1 in liver led to progressive kidney fibrosis in mice (Kopp et al., 1996), whereas overexpression of latent TGF-β1 in the skin displayed anti-inflammatory and anti-fibrosis effects in obstructive and crescentic glomerulonephritis (Huang et al., 2008a, b). The distinct functions of active and latent TGF-β1 in renal fibrosis and inflammation suggest that a better therapeutic approach would be to block conversion of latent TGF-β to active TGF-β. Wong et al. (2011) showed that inhibiting conversion of latent to active TGF-β1 in human proximal tubular cells reduced matrix protein expression and inhibited fibrosis under hyperglycemia and hypoxia conditions. What is more, the αv-containing integrins with different β-subunits that interact with latent TGF-β1 and activate TGF-β1 have a critical function in kidney fibrosis. A pharmacologic inhibitor of αvβ1 integrin prevented activation of the latent TGF-β complex and ameliorated renal fibrosis in mice fed an adenine diet (Chang et al., 2017). The mechanisms of the distinct functions of latent versus active TGF-β1 may be related to the prevention of Smad7 from Smurf-mediated ubiquitination and degradation in response to higher levels of latent TGF-β1 (Lan, 2011). Smad7 inhibits TGF-β signaling by promoting degradation of the TβRI and inhibiting Smad2/3/4 activity (Nakao et al., 1997a; Miyazawa and Miyazono, 2017). But in chronic kidney disease, active TGF-β1 activates the Smurfs and arkadia-dependent ubiquitin-proteasome pathways, which degrades Smad7 protein by a post-transcriptional modification mechanism (Kavsak et al., 2000; Ebisawa et al., 2001; Fukasawa et al., 2004).

## Conclusion

On the basis of experimental and clinical studies, modulating TGF-β1, instead of directly inhibiting TGF-β1 ligands/receptors, may be a good antifibrosis tactic for DKD. TGF-β1 promotes wound healing (Wang et al., 2014), tissue regeneration (Borges et al., 2013), anti-inflammation (Kulkarni et al., 1993), autophagy (Koesters et al., 2010), and urinary glucose regulation (Hathaway et al., 2015). Nonetheless, the dose regimen must be considered carefully because a large dose of TGF-β blockade had severe toxicity and poor efficacy in animal experiments (Khanna et al., 2004; Ma et al., 2004). A pan-neutralizing monoclonal antibody could also lead to undesired effects such as tumor formation, even though animal studies have not exhibited such events during prolonged TGF-β1 inhibition. What is more, developing molecules that suppress the activation of latent TGF-β1 would be a potential therapy. Given the central role of TGF-β1 in the pathophysiology of DKD, the TGF-β1 system is an attractive target to retard the progression of DKD, provided that the approach maintains an acceptable balance between renoprotective and harmful effects.

## Author Contributions

LZ and FL conceptualized this review and decided on the content. LZ, YZ, and FL wrote and revised the manuscript. All authors approved the final version of the manuscript.

## Conflict of Interest

The authors declare that the research was conducted in the absence of any commercial or financial relationships that could be construed as a potential conflict of interest.
